# Effectiveness of autologous emulsified stromal vascular fraction tissue injection for the treatment of complex perianal fistulas in inflammatory bowel diseases patients: a pilot study

**DOI:** 10.1177/17562848241263014

**Published:** 2024-09-04

**Authors:** Angelo Eugenio Potenza, Dania Nachira, Franco Sacchetti, Angelo Trivisonno, Daniela Pugliese, Ivo Boškoski, Giuseppe Caudullo, Laura Maria Minordi, Luigi Larosa, Paola Caprino, Franco Scaldaferri, Luigi Sofo, Venanzio Porziella

**Affiliations:** Abdominal Surgery Unit, Department of Gastroenterological, Endocrine-Metabolic and Nephro-Urological Sciences, Fondazione Policlinico Universitario ‘A. Gemelli’ IRCCS, Rome, Italy; Department of General Thoracic Surgery, Fondazione Policlinico Universitario ‘A. Gemelli’ IRCCS, Università Cattolica del Sacro Cuore, Roma, Italy; Abdominal Surgery Unit, Department of Gastroenterological, Endocrine-Metabolic and Nephro-Urological Sciences, Fondazione Policlinico Universitario ‘A. Gemelli’ IRCCS, L. go A. Gemelli 8, Rome 00168, Italy; Unit of Plastic Surgery, Assunzione di Maria Santissima Clinic, Rome, Italy; IBD Unit, CEMAD Centro Malattie dell’Apparato Digerente, Medicina Interna e Gastroenterologia Fondazione Policlinico Universitario Gemelli IRCCS, Roma, Italy; Dipartimento di Medicina e Chirurgia Traslazionale, Università Cattolica del Sacro Cuore, Roma, Italy; Center for Endoscopic Research Therapeutics and Training, Università Cattolica del Sacro Cuore, Rome, Italy; Digestive Endoscopy Unit, Fondazione Policlinico Universitario ‘A. Gemelli’ IRCCS, Rome, Italy; School of General Surgery, Università Cattolica del Sacro Cuore, Roma, Italy; Department of Diagnostic Imaging, Oncological Radiotherapy, and Hematology, Fondazione Policlinico Universitario ‘A. Gemelli’ IRCCS, Rome, Italy; Department of Diagnostic Imaging, Oncological Radiotherapy, and Hematology, Fondazione Policlinico Universitario ‘A. Gemelli’ IRCCS, Rome, Italy; Abdominal Surgery Unit, Department of Gastroenterological, Endocrine-Metabolic and Nephro-Urological Sciences, Fondazione Policlinico Universitario ‘A. Gemelli’ IRCCS, Rome, Italy; IBD Unit, CEMAD Centro Malattie dell’Apparato Digerente, Medicina Interna e Gastroenterologia Fondazione Policlinico Universitario Gemelli IRCCS, Roma, Italy; Dipartimento di Medicina e Chirurgia Traslazionale, Università Cattolica del Sacro Cuore, Roma, Italy; Abdominal Surgery Unit, Department of Gastroenterological, Endocrine-Metabolic and Nephro-Urological Sciences, Fondazione Policlinico Universitario ‘A. Gemelli’ IRCCS, Università Cattolica del Sacro Cuore, Roma, Italy; Department of General Thoracic Surgery, Fondazione Policlinico Universitario ‘A. Gemelli’ IRCCS, Università Cattolica del Sacro Cuore, Roma, Italy

**Keywords:** fistula, inflammatory bowel disease, mesenchymal stromal cells

## Abstract

Complex fistulizing perianal disease is a disabling manifestation of inflammatory bowel disease (IBD), seriously compromising patients ‘quality of life’. The success rate of available treatments is quite low, and nearly half of the patients will develop chronically active fistulas or experience fistula recurrence. Mesenchymal stem cell therapy has shown interesting results, but the complexity and the cost of production limit its widespread use. This study aims to report the results of the innovative use of autologous emulsified adipose-derived stromal vascular fraction tissue for treating complex fistulizing perianal disease. From March 2021 to March 2022, 10 patients underwent a two-step procedure: (1) examination under anaesthesia, with loose seton drainage and 4 weeks later and (2) curettage of the fistulous tract, internal fistula closure and an injection of autologous emulsified adipose-derived stromal vascular fraction tissue harvested from the subcutaneous layer of the patient’s hip. Clinical and radiological (through magnetic resonance imaging) healing were assessed at 6 months. We included five patients affected by Crohn’s disease, three by ulcerative colitis and two by indeterminate colitis. All patients were on concomitant biological therapy (50% on Infliximab). One patient required a re-treatment for a relapse and two different fistulas were separately treated in another one. Out of 12 total procedures performed, clinical healing was achieved in 10 cases (83%), while radiological healing in 6 patients (50%). No adverse events were recorded. Autologous emulsified adipose-derived stromal vascular fraction tissue can represent an effective, safe and cheap add-on therapy for patients with complex perianal fistulas in IBDs.

## Introduction

Complex perianal fistulas represent one of the most disabling complications of inflammatory bowel disease (IBD), involving up to 28% of patients affected by Crohn’s disease^1^ (CD) within the first 20 years from diagnosis.^2^ Standard of care is represented by a multidisciplinary approach, with a first surgical stage for controlling sepsis and seton placement (±antibiotics), followed by a biological therapy with infliximab.^3^

Conventional surgical treatments for fistula closure, sometimes inherited from cryptoglandular fistula surgery, are often not completely effective in IBD patients’ and are burdened by high rates of faecal incontinence with a significant impact on their quality of life (QoL).^4^

Encouraging results, however, have been obtained in recent decades thanks to minimally invasive surgery often associated with local cell/tissue therapies.^5^

In particular, add-on topical therapy with allogenic mesenchymal stem cells (MSCs; multipotent adult stem cells) was shown to increase the rate of combined fistula remission (defined as clinical plus radiological through magnetic resonance imaging, MRI) in up to 50% of patients after 52 weeks with a good safety profile.^6,7^

Despite these encouraging results, given in a subset of patients with limited therapeutic options, several barriers limit patients’ treatment access in clinical practice, mainly due to stringent inclusion criteria (such as only patients with a CD diagnosis, mild-quiescent clinical activity, rectal sparing and no diverting stoma) and costs.

Therefore, autologous MSCs therapy represents a novel alternative therapeutic option.^8,9^ However, most of the methods proposed for obtaining autologous MSCs from bone marrow or adipose tissue are based on three main steps, that is (1) cell harvesting, (2) isolation through enzymatic or chemical processes and expansion in cell factory and (3) administration to patients.

In recent years, some experiences have been reported so far on the use of MSCs derived from autologous adipose tissue and extracted by purely mechanical methods such as those obtained from the disposable device Lipogems^®^ (Lipogems International SpA, Milan, Italy) kit, reducing progressively in size the clusters of adipose tissue.^10^

Lipogems was employed by Naldini *et al.*^11^ in a trial involving complex idiopathic fistula-in-ano and subsequently by Laureti *et al.*^12^ in 2020 for 15 patients with CD-related complex perianal fistulas, refractory to biological therapy with encouraging results in both settings.

In this paper, we present our experience with the innovative use of autologous adipose-derived stromal vascular fraction emulsified tissue (tSVFem) rich in MSCs^13,14^ for the treatment of complex perianal fistulas in patients affected by IBD.

## Materials and methods

The reporting of this study conforms to the CARE Guidelines: Consensus-based Clinical Case Reporting Guideline Development.^15^

We included 10 patients with a confirmed diagnosis of IBD with active complex fistulizing perianal disease, and failure to standard multimodal treatments after at least 1 year of continuous biological therapy. The definition of complex fistulizing perianal disease from the American Gastroenterological Association^16^ was adopted for all patients, even though this classification is specifically for perianal lesions occurring in patients affected by CD.

Clinical disease activity was assessed using the Harvey–Bradshaw index for CD and the partial Mayo score (MS) for ulcerative colitis (UC). Endoscopic activity was assessed using the Simple Endoscopic Score for Crohn’s disease for CD and the endoscopic MS for UC.

**Figure 1.**
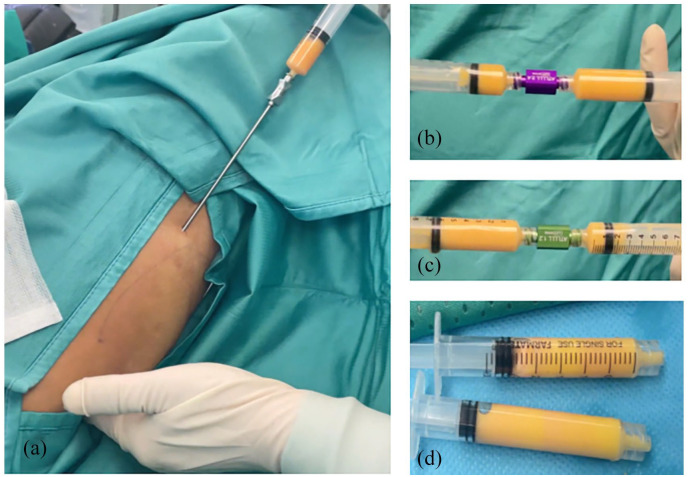
(a) Aspiration of adipose tissue, (b, c) Filtration after centrifugation and (d) Final tSVFem. tSVFem, stromal vascular fraction emulsified tissue.

According to standard procedure, all patients, after an accurate diagnosis, and classification through pelvic MRI, underwent examination under anaesthesia, with loose seton drainage. Four weeks after the first surgical step, an injection of autologous tSVFem was scheduled. All procedures were performed with patients in lithotomy position and laryngeal mask with deep sedation. After rectal exploration, the absence of further fistulous tracts or unacknowledged abscesses was verified. By injecting saline solution through the external fistulous orifice, patency of the fistulous tract was verified and the internal fistulous orifice was identified using an Eisenhammer retractor. Curettage of the fistulous tract was performed by Volkmann’s spoon to remove the granulation tissue and stimulate the regenerative response. The internal fistulous orifice was then closed using stitches in resorbable filament, and the integrity of the suture was verified by further injection of saline solution from the fistula. At the same time, 30 ml of autologous fat was harvested from the subcutaneous layer of the patient’s hip using a specially designed 2.1 mm micro-cannula. Twenty millilitres of autologous ‘microfat’ was emulsified by sequential filters and centrifuged (at 3000 rounds for 3 min) to remove oil and liquid fractions. The detailed procedure was already described in our previous report^13,14^ and outlined in Figure 1 and shown in the Supplemental Material.

**Figure 2.**
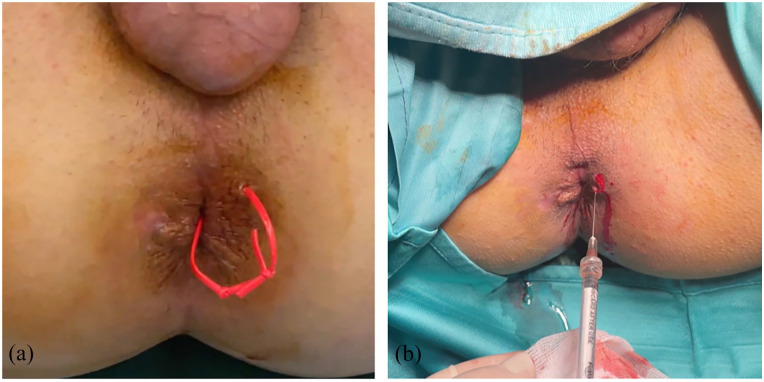
(a) Preoperative inspection and (b) tSVFem administration along the fistulous tract. tSVFem, stromal vascular fraction emulsified tissue.

The process of obtaining the tSVFem involves exclusively mechanical methods without using drugs, enzymatic reactions or other chemical substances. The tSVFem so obtained was injected around the border of the previously closed internal fistulous orifice and along the course of the fistulous tract through a 22-gauge needle (Figure 2).

Perianal Disease Activity Index (PDAI), Cleveland Global Quality of Life (CGQL), Quality of Life (QoL) section of CGQL, Quality of Health (QoH) of CGQL and Energy Level (EL) of CGQL^17^ were recorded for each patient at baseline and 6 months after treatment. The primary outcome was clinical fistula healing, defined as the closure of all treated external opening(s) and no drainage after gentle finger compression at 6 months.

Secondary outcomes were as follows: (1) radiological fistula healing, defined as the absence of perianal abscesses >3 mm or fluid in the tract of treated fistula(s) at MRI; (2) predictors of clinical healing; and (3) improvement from baseline in PDAI, CGQL, QoL, QoH and EL scores assessed at 6 months.

The study was conducted in accordance with the ethical standards of the Declaration of Helsinki and every detail of the patients involved in the study was de-identified.

Statistical analysis was performed using SPSS software (v. 24.0; Inc., Chicago, IL, USA). Data were expressed as means ± standard deviation (SD) and median with data range. Pearson χ^2^ test and Fischer’s exact test were used to compare discrete variables, and Student’s *t*-test to compare means between two continuous variables.

## Results

Ten patients were enrolled and treated between March 2021 and March 2022: six patients affected by CD, three by UC and one patient affected by pouch-perianal fistula (occurred 18 years after pouch surgery). The mean age was 33.6 years (SD ± 13.4), and the mean duration of the disease was 12.4 years (SD ± 9.3). All patients were on concomitant biological therapy (50% on infliximab, 20% vedolizumab, 20% adalimumab, and 10% ustekinumab) for a median duration of 27.2 months (SD ± 26.1).

Eight patients had trans-sphincteric fistulas, one extra-sphincteric and one inter-sphincteric.

Each patient had previously undergone partial fistulectomies with the placement of loose setons without any benefit. No patient had undergone previous fistula repair techniques such as mucosal flap or ligation of the intersphincteric fistulous tract (LIFT).

Table 1 summarizes baseline patients’ characteristics.

**Table 1.** Clinical characteristics of patients treated.

Patient	Sex	Age	Disease	Duration of disease (years)	Number of fistulas	HBI	PMS	SES-CD	MS	Rectal disease activity	Park’s classification	Systemic therapy
1	F	23	CD	11	1	2	/	0	/	N	Transsphincteric	Infliximab
2	M	41	UC	20	1	/	0	/	1	Y	Extrasphincteric	Vedolizumab
3	M	44	UC W/IPAA	25	1	/	2	/	1	Y	Transsphincteric	Adalimumab
4	M	31	CD	23	4	1	/	9	/	N	Transsphincteric	Ustekinumab
5	M	26	UC	7	1	/	1	/	1	Y	Intersphincteric	Infliximab
6	M	21	CD	5	1	2	/	4	/	N	Transsphincteric	Infliximab
7	F	22	CD	2	1	2	/	4	/	N	Transsphincteric	Adalimumab
8	M	64	CD	5	1	6	/	6	/	N	Transsphincteric	Vedolizumab
9	M	27	UC	3	1	/	2	/	1	N	Transsphincteric	Infliximab
10	F	37	CD	23	2	2	/	4	/	Y	Transsphincteric	Infliximab

CD, Crohn’s disease; HBI, Harvey–Bradshaw index; IPAA, ileal pouch-anal anastomosis; MS, Mayo score; PMS, partial Mayo score; SES-CD, Simple Endoscopic Score for Crohn’s disease; UC, ulcerative colitis.

Two patients underwent a double procedure of administration of tSVFem: the first one for fistula recurrence 11 months after the study procedure and the second one for treating two different fistulas after 7 months. The follow-up was set at 6 months after each procedure.

**Figure 3.**
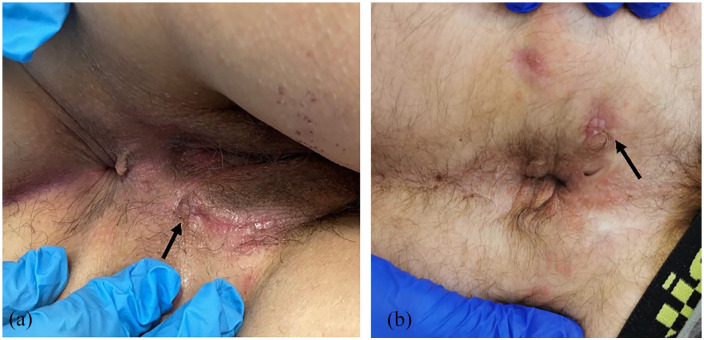
(a, b) Clinical healing at 6 months after the procedure in two different patients.

**Figure 4.**
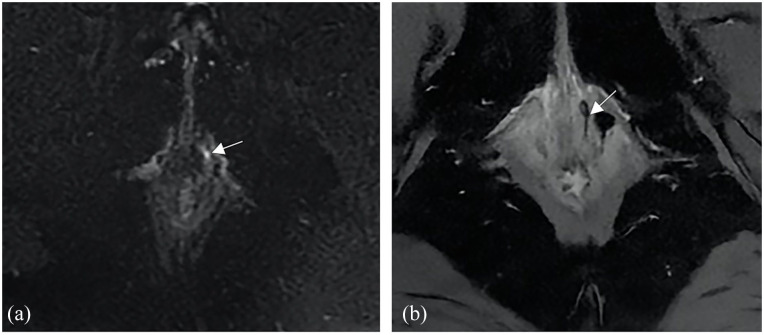
(a) Axial T2-weighted fat-suppressed image shows an intersphincteric fistula with fluid content in the left anterolateral wall of the anal canal (arrow). (b) Axial T2-weighted fat-suppressed image shows the resolution of the fluid content in the fistula (arrow) after the surgical procedure.

Overall, out of 12 total procedures performed, clinical healing was achieved in 10 cases (83.3%), while radiological healing was in 6 patients (50%) at 6 months (Figures 3 and 4) as summarized in Table 2.

**Table 2.** Clinical, radiological healing and relative QoL indexes for each procedure.

Procedure	Clinical healing	Radiological healing	Preoperative PDAI	Postoperative PDAI	Preoperative QoL	Postoperative QoL	Preoperative CGQL	Postoperative CGQL
1	Yes	Yes	5	4	8	10	23	26
2^a^	No	No	4	4	6	7	18	21
3^a^	Yes	No	4	4	7	8	22	24
4^b^	Yes	Yes	4	4	6	6	20	20
5^b^	Yes	Yes	5	4	6	6	18	19
6	Yes	No	4	5	2	8	27	25
7	Yes	Yes	7	6	7	7	19	25
8	Yes	No	6	3	8	10	25	30
9	No	No	5	5	2	4	20	22
10	Yes	Yes	7	6	6	8	20	24
11	Yes	Yes	3	2	8	9	26	27
12	Yes	No	3	3	7	8	24	25

a Patient 2.

b Patient 3.

CGQL, Cleveland Global Quality of Life; PDAI, Perianal Disease Activity Index; QoL, quality of life.

Regarding the two patients who did not achieve clinical healing, the first one, as previously reported, was retreated with a second administration of tSVFem 11 months later, resulting in fistula closure. Conversely, the second one had persistent secretions from the treated fistula and radiological evidence of the persistence of fluid in the fistulous tract.

The median duration of the study procedure was 73 min (range 43–119). No short- and long-term adverse events were recorded. There was no correlation between clinical healing and type of IBD disease (*p* = 0.687), sex (*p* = 0.371), age (*p* = 0.419), radiological healing (*p* = 0.121) and concomitant therapies (*p* = 0.126).

Six-month QoL scores significantly increased in patients who achieved clinical fistula healing compared to baseline ones (8.25 ± 1.39 *versus* 7.00 ± 0.92, *p* = 0.05). Conversely, no differences emerged in terms of QoH (*p* = 0.839), EL (*p* = 0.699) or CGQL (*p* = 0.402).

## Discussion

Treatment of complex perianal fistulas remains one of the biggest challenges in managing patients affected by IBD, with low therapeutic success rates and a significant impact on QoL. Therapy with allogeneic MSCs is safe since a very low risk of systemic side effects and faecal incontinence compared to advanced surgical procedures (such as advancement flap or LIFT).^6^

The long-term results (104 weeks) from the largest trial on the use of allogeneic stem cells have documented a combined healing rate in the experimental group of 56% compared to 40% of the control group.^18^

However, this treatment is currently reserved only for a specific subsetting of patients affected by CD and it is burdened by important production and management costs, not widely sustainable by healthcare systems.

Furthermore, as documented by Cheng *et al.*^19^ in a 2019 systematic review and meta-analysis, the allogeneic origin of MSCs does not appear to provide better outcomes in terms of clinical or radiological healing compared to autologous MSCs in the treatment of fistulizing perianal disease in IBD.

In this report, we presented results of the first applications of autologous tSVFem rich in MSCs (already in use at our centre for the management of oesophageal fistulas^13^) for the treatment of 10 cases of complex IBD-related perianal fistula. We included patients who failed standard multimodal treatment, in particular biological therapies, regardless of the underlying IBD diagnosis.

Overall, of 12 procedures performed, 10 (83.3%) resulted in clinical fistula healing (Figure 3) and 6 (50%) in radiological healing at 6 months (Figure 4). Moreover, a significant improvement in QoL score was recorded among patients achieving clinical fistula healing. No specific baseline clinical variables have been identified predicting fistula healing.

We did not record any short- or long-term study procedure adverse events.

This could be mainly related to the autologous nature of the product, an aspect that substantially differentiates the tSVFem from allogeneic MSCs. Indeed, the absence of enzymatic and chemical reactions and purifications keeps unchanged the natural cellular secretoma, with its anti-inflammatory and immunomodulatory potentials, adding proangiogenic and regenerative functions to tSVFem, improving tissue healing and reducing the inflammatory risks.

The use of autologous adipose tissue for MSCs extraction has already been studied in literature in the treatment of complex fistulizing IBD-related perianal disease.

In 2020, an open-label controlled trial was published by Zhou *et al.*^20^ enrolling 22 patients with CD-related complex perianal fistulas treated with MSCs from autologous adipose tissue manipulated by enzymatic processes and inoculated into a culture flask for later administration.

At 12 months, 63.6% of patients in the treatment group achieved combined remission (clinical and MRI/endorectal ultrasonography) *versus* 54.5% of controls who underwent drainage only and surgical closure of the internal fistulous orifice.

The method proposed by Zhou *et al.* has, in our opinion, two main limitations, which are the application of enzymatic methods for the purification of MSCs and the requirement of *in vitro* cultivation procedures. Accordingly, a second surgical time for the administration of cell therapy is required, increasing the length of treatment timing and the logistical costs.

Conversely, our procedure is inexpensive and sustainable because (1) to obtain tSVFem, only mechanical manipulation of tissue through sequential filters and centrifugation is required, without the necessity of a cell factory or enzymatic substances; (2) the collection, the extraction and the administration of tSVFem can be performed in the same operative session (median duration of the entire procedure: 73 min, range 43–119) and (3) the production costs are also meagre since the cannula used for harvesting subcutaneous adipose tissue and other instruments used for obtaining tSVFem can be reused.

Moreover, the procedure can be easily performed in a day-surgery mode without requiring patient hospitalization.

In 2020 Laureti *et al.*^12^ published the effectiveness data on 15 patients affected by refractory complex fistulizing perianal CD, treated with autologous MSCs, mechanically processed through the Lipogems kit, a special disposable device, reducing progressively in size the clusters of adipose tissue with the elimination of oil and blood residues. At 24 weeks, combined remission (defined as clinical plus radiographic) was recorded in 10 patients, improvement in 4 and failure only in 1 patient.

These encouraging data may pave the way for a new promising application of the Lipogems system, which is already in use in other fields (e.g. orthopaedics), for the treatment of CD.

Compared with Lipogems system, our instruments for the production of tSVFem consist exclusively of sterilizable and reusable filters and cannulas without the need for disposable kits, representing a more feasible and sustainable approach.

Moreover, we decided to not restrain only CD patients, but including also patients affected by UC and with pouch-perianal fistula. This aspect can alternatively represent a strength point, expanding the application to more patients, but also a weakness, further reducing the sample size for each specific setting.

At last, the absence of a control group represents another important limitation of our study.

Given the above limitations, autologous tSVFem seems to be safe and cheap and it has the potential to be included in the armoury in the treatment of IBD-related complex perianal fistulas.

These initial encouraging results can represent a starting point for future studies with a larger population and longer follow-up period, to assess long-term effectiveness and safety.

## Supplemental Material

sj-pdf-1-tag-10.1177_17562848241263014 – Supplemental material for Effectiveness of autologous emulsified stromal vascular fraction tissue injection for the treatment of complex perianal fistulas in inflammatory bowel diseases patients: a pilot study Supplemental material, sj-pdf-1-tag-10.1177_17562848241263014 for Effectiveness of autologous emulsified stromal vascular fraction tissue injection for the treatment of complex perianal fistulas in inflammatory bowel diseases patients: a pilot study by Angelo Eugenio Potenza, Dania Nachira, Franco Sacchetti, Angelo Trivisonno, Daniela Pugliese, Ivo Boškoski, Giuseppe Caudullo, Laura Maria Minordi, Luigi Larosa, Paola Caprino, Franco Scaldaferri, Luigi Sofo and Venanzio Porziella in Therapeutic Advances in Gastroenterology

## Declarations

**Ethics approval and consent to participate:** The study was approved by the local Ethics Committee of Università Cattolica del Sacro Cuore, Rome, Italy (Prot ID 3127, 2020) and all patients provided written informed consent to treatment.

**Consent for publication:** Not applicable.

**Availability of data and materials:** The data underlying this article will be shared on reasonable request to the corresponding author.
