# Supplementary material for: Effectiveness of autologous emulsified stromal vascular fraction tissue injection for the treatment of complex perianal fistulas in inflammatory bowel diseases patients: a pilot study
Source: Ther Adv Gastroenterol. 2024 Sep 4;17:17562848241263014. doi: 10.1177/17562848241263014 (PMC11375657; doi:10.1177/17562848241263014)
Supplement: sj-pdf-1-tag-10.1177_17562848241263014 – Supplemental material for Effectiveness of autologous emulsified stromal vascular fraction tissue injection for the treatment of complex perianal fistulas in inflammatory bowel diseases patients: a pilot study [file sj-pdf-1-tag-10.1177_17562848241263014.pdf]

CARE Checklist of information to include when writing a case report

CARE case report guidelines

(cc) BY-NC-ND

Topic	Item	Checklist item description	Reported on Line
**Title**	1	The diagnosis or intervention of primary focus followed by the words "case report"	
**Key Words**	2	2 to 5 key words that identify diagnoses or interventions in this case report, including "case report"	149
**Abstract (no references)**	3a	Introduction: What is unique about this case and what does it add to the scientific literature?	119
	3b	Main symptoms and/or important clinical findings	133
	3c	The main diagnoses, therapeutic interventions, and outcomes	136
	3d	Conclusion—What is the main "take-away" lesson(s) from this case?	139
**Introduction**	4	One or two paragraphs summarizing why this case is unique (**may include references**)	222
**Patient Information**	5a	De-identified patient specific information.	274
	5b	Primary concerns and symptoms of the patient.	229
	5c	Medical, family, and psycho-social history including relevant genetic information	237
	5d	Relevant past interventions with outcomes	300
**Clinical Findings**	6	Describe significant physical examination (PE) and important clinical findings.	299
**Timeline**	7	Historical and current information from this episode of care organized as a timeline	
**Diagnostic Assessment**	8a	Diagnostic testing (such as PE, laboratory testing, imaging, surveys).	308
	8b	Diagnostic challenges (such as access to testing, financial, or cultural)	
	8c	Diagnosis (including other diagnoses considered)	
	8d	Prognosis (such as staging in oncology) where applicable	
**Therapeutic Intervention**	9a	Types of therapeutic intervention (such as pharmacologic, surgical, preventive, self-care)	239
	9b	Administration of therapeutic intervention (such as dosage, strength, duration)	
	9c	Changes in therapeutic intervention (with rationale)	
**Follow-up and Outcomes**	10a	Clinician and patient-assessed outcomes (if available)	307
	10b	Important follow-up diagnostic and other test results	324
	10c	Intervention adherence and tolerability (How was this assessed?)	324
	10d	Adverse and unanticipated events	359
**Discussion**	11a	A scientific discussion of the strengths AND limitations associated with this case report	424
	11b	Discussion of the relevant medical literature **with references**.	330
	11c	The scientific rationale for any conclusions (including assessment of possible causes)	
	11d	The primary "take-away" lessons of this case report (without references) in a one paragraph conclusion	429
**Patient Perspective**	12	The patient should share their perspective in one to two paragraphs on the treatment(s) they received	
**Informed Consent**	13	Did the patient give informed consent? Please provide if requested	Yes ☒ No ☐
